# Supplementary material for: A universal method for generating knockout mice in multiple genetic backgrounds using zygote electroporation
Source: Biol Open. 2023 Sep 6;12(9):bio059970. doi: 10.1242/bio.059970 (PMC10497038; doi:10.1242/bio.059970)
Supplement: Supplementary information [file biolopen-12-059970-s1.pdf]

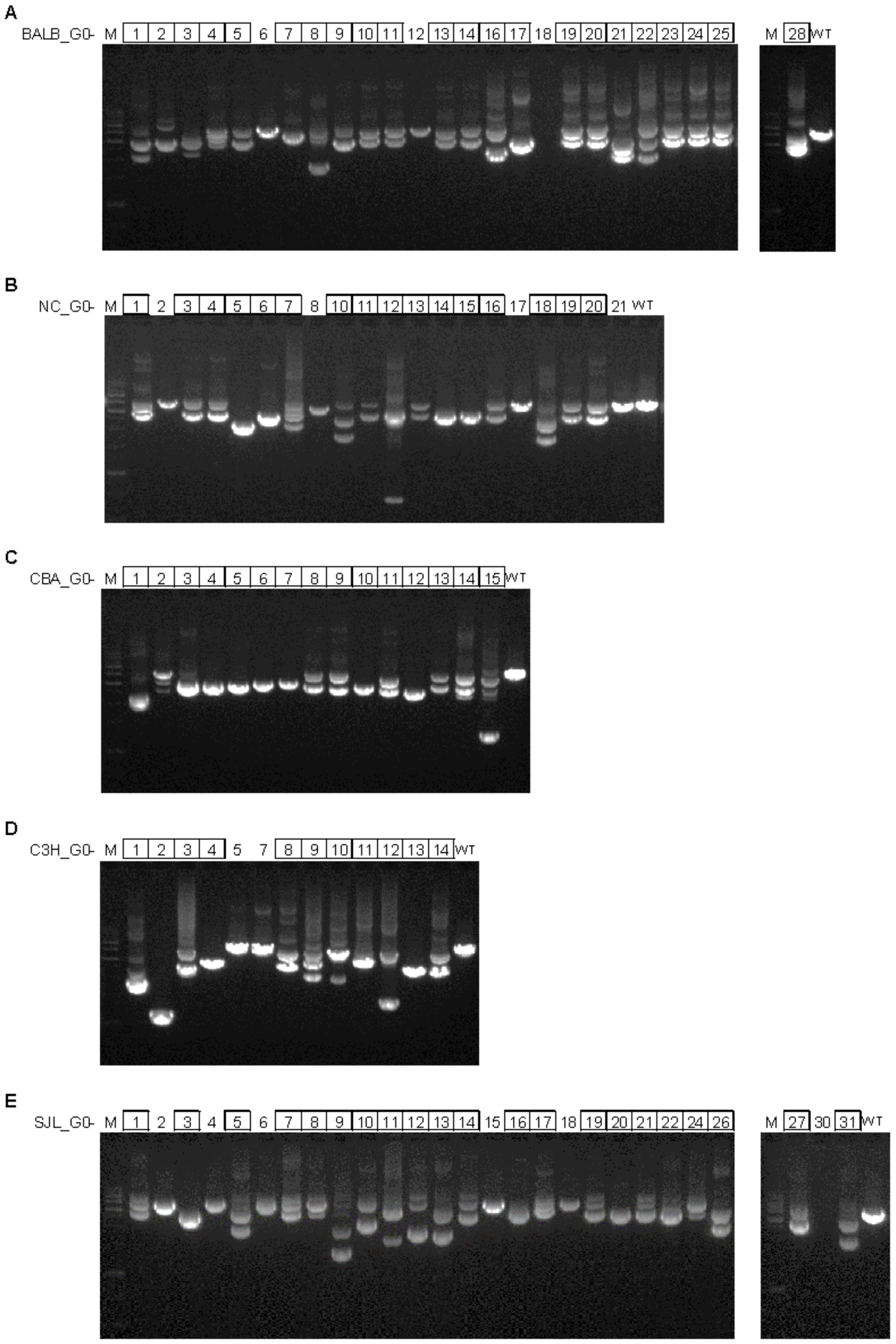

Biology Open • Supplementary information

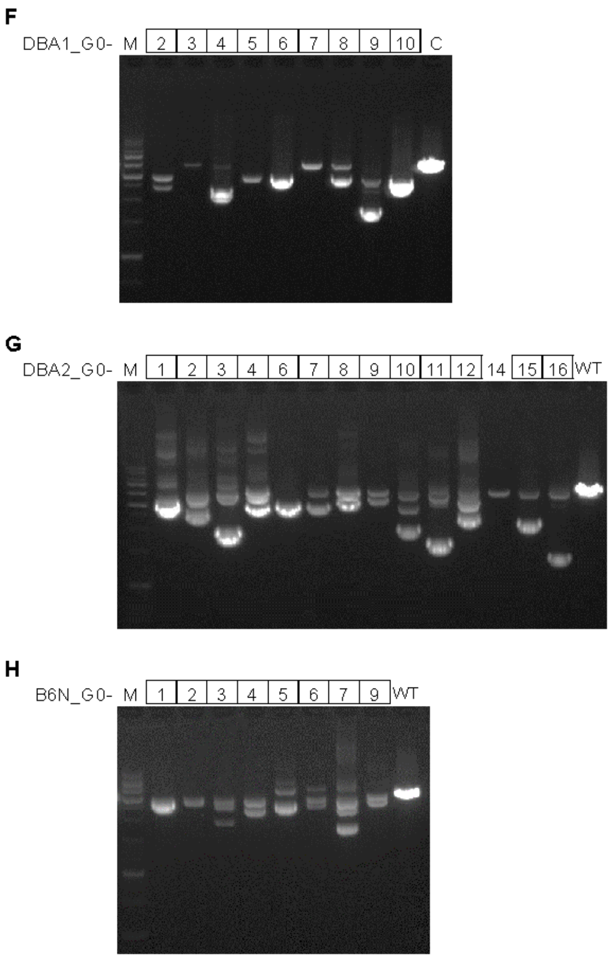

Fig. S1. Electrophoresis results of long-PCR in G0 mice

Numbers indicate mouse IDs, and black boxes indicate mice subjected to long-read analysis owing to the intended loss of target exons. WT represents wild-type mice of each inbred strain.

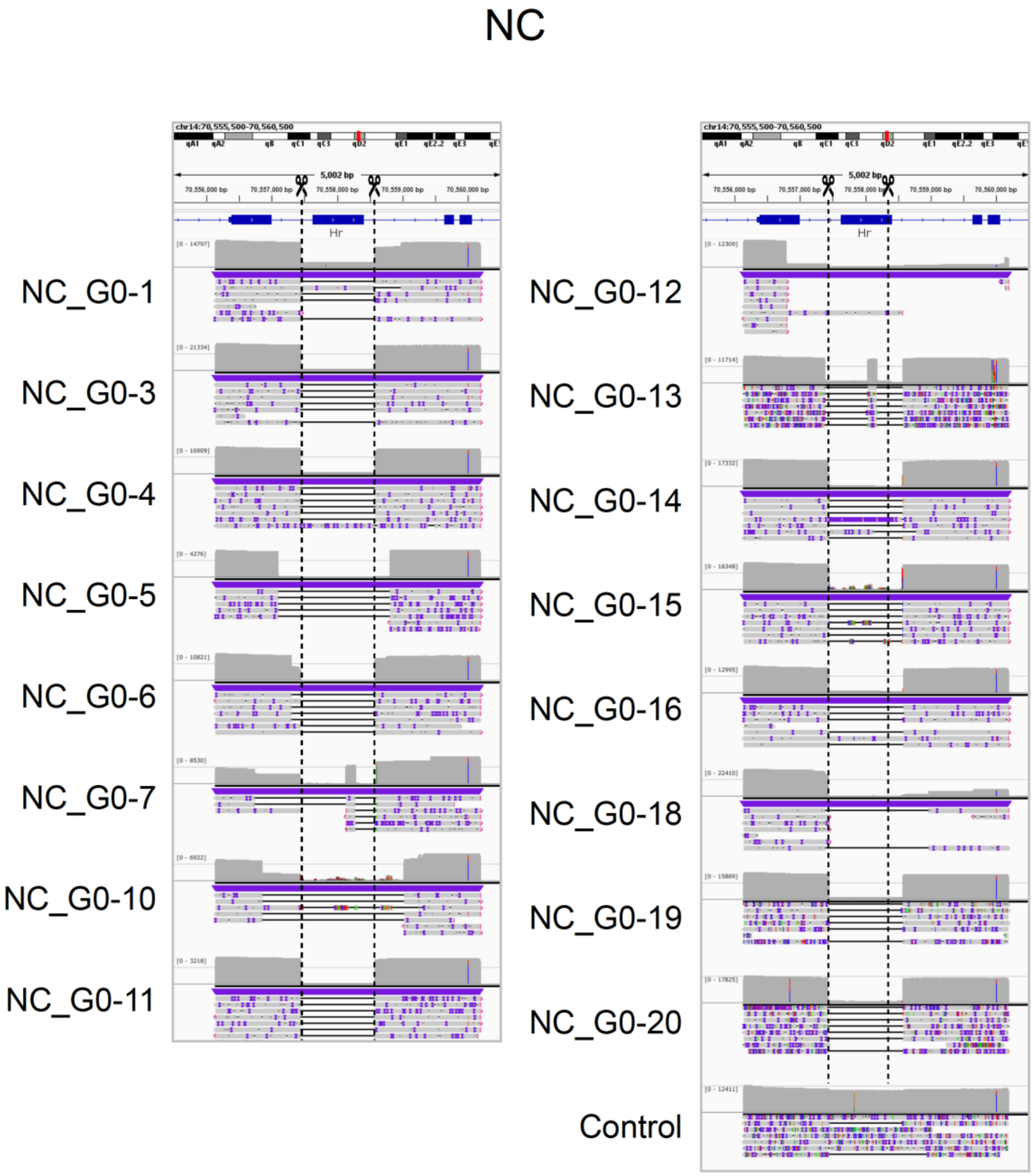

Biology Open • Supplementary information

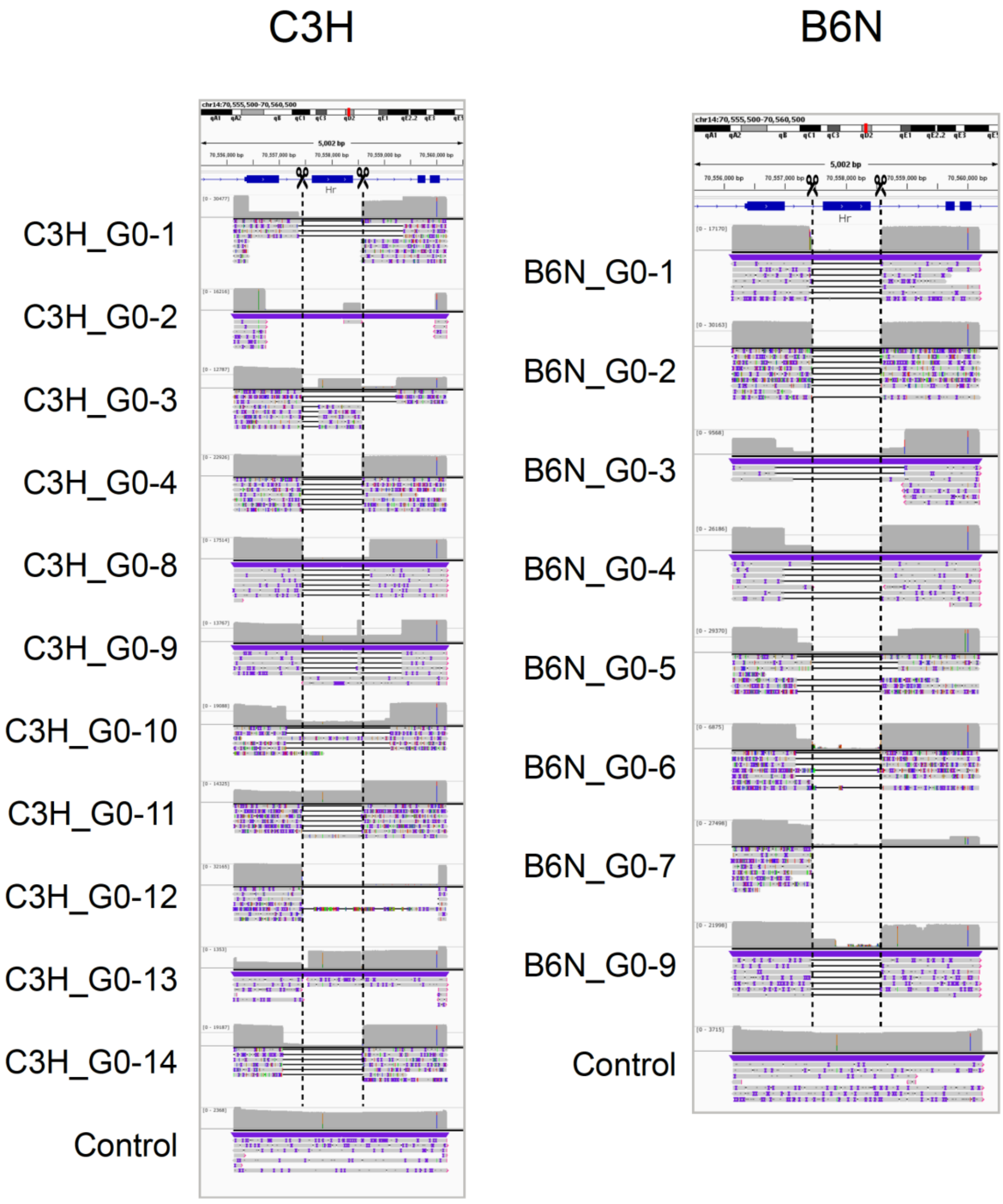

Fig. S2. Visualisation of nanopore sequence reads of NC, C3H, and B6N at the *Hr* target locus.

Visualisation of nanopore sequence reads at the *Hr* target locus in GRCm38.p6.

Scissors and dotted lines represent cleavage sites predicted by Cas9.

Biology Open • Supplementary information

Table S1. Survival and re-derivation rate of electroporated B6N zygotes in parallel experiments.

Strain	# of examined zygotes[A]	# of survived zygotes[B] (B/A)	# of embryos developed to two-cell stage[C] (C/B)	# of transferred two-cell embryos[D]	# of recipients[E]	# of pregnancy[F] (F/E)	# of newborns[G] (G/D)
B6N	186	186 (100%)	181 (97.3%)	174	6	6 (100.0%)	61 (35.1%)

#, number.

Table S2. Details of the alleles in G1 mice.

Click here to download Table S2

Table S3. Age of female mice for super-ovulation and results of retired breeders.

Strain	Age of females	# of examined females[A]	# of super-ovulated females[B] (B/A)	# of oocytes[C]	# of zygotes[D] (D/C)
BALB/c	11wks	12	12 (100.0%)	204	195 (95.6%)
	Retired breeders	15	12 (80.0%)	117	115 (98.3%)
NC	9-10 wks				
CBA	9-11 wks				
C3H	11 wks				
SJL	9 wks				
DBA1	11wks	12	12 (100.0%)	268	186 (69.4%)
	Retired breeders	12	11 (91.7%)	242	242 (100.0%)
DBA2	9-10 wks				
B6N	9-11 wks				

Age of retired females ranged from 8-weeks- to approximately 12-months-old. #, number.

Biology Open • Supplementary information

Table S4. Genomic DNA extraction methods in G0 mice.

Click here to download Table S4

Table S5. Primer sequences used for long-read and short-read sequencing.

Click here to download Table S5

Biology Open • Supplementary information
